# Interaction between nectin-1 and the human natural killer cell receptor CD96

**DOI:** 10.1371/journal.pone.0212443

**Published:** 2019-02-13

**Authors:** Veronica M. Holmes, Carlos Maluquer de Motes, Paige T. Richards, Jessenia Roldan, Arjun K. Bhargava, Jordan S. Orange, Claude Krummenacher

**Affiliations:** 1 Department of Biochemistry, School of Dental Medicine, University of Pennsylvania, Philadelphia, Pennsylvania, United States of America; 2 Department of Microbial Sciences, University of Surrey, Guildford, Surrey, United Kingdom; 3 Department of Biological Sciences, Rowan University, Glassboro, New Jersey, United States of America; 4 Department of Pediatrics, Columbia University, New York, New York, United States of America; 5 Department of Molecular and Cellular Biosciences, Rowan University, Glassboro, New Jersey, United States of America; Universiteit Gent, BELGIUM

## Abstract

Regulation of Natural Killer (NK) cell activity is achieved by the integration of both activating and inhibitory signals acquired at the immunological synapse with potential target cells. NK cells express paired receptors from the immunoglobulin family which share common ligands from the nectin family of adhesion molecules. The activating receptor CD226 (DNAM-1) binds to nectin-2 and CD155, which are also recognized by the inhibitory receptor TIGIT. The third receptor in this family is CD96, which is less well characterized and may have different functions in human and mouse models. Human CD96 interacts with CD155 and ligation of this receptor activates NK cells, while in mice the presence of CD96 correlates with decreased NK cell activation. Mouse CD96 also binds nectin-1, but the effect of this interaction has not yet been determined. Here we show that human nectin-1 directly interacts with CD96 *in vitro*. The binding site for CD96 is located on the nectin-1 V-domain, which comprises a canonical interface that is shared by nectins to promote cell adhesion. The affinity of nectin-1 for CD96 is lower than for other nectins such as nectin-3 and nectin-1 itself. However, the affinity of nectin-1 for CD96 is similar to its affinity for herpes simplex virus glycoprotein D (HSV gD), which binds the nectin-1 V-domain during virus entry. The affinity of human CD96 for nectin-1 is lower than for its known activating ligand CD155. We also found that human erythroleukemia K562 cells, which are commonly used as susceptible targets to assess NK cell cytotoxicity did not express nectin-1 on their surface and were resistant to HSV infection. When expressed in K562 cells, nectin-1-GFP accumulated at cell contacts and allowed HSV entry. Furthermore, overexpression of nectin-1-GFP led to an increased susceptibility of K562 cells to NK-92 cell cytotoxicity.

## Introduction

Nectins and nectin-like (Necl) proteins are cell adhesion molecules from the immunoglobulin (Ig) superfamily, which are characterized by three extracellular Ig domains [1]. Nectins and Necls (further referred to collectively as nectins) play important roles in development and tissue organization, notably in the CNS and epithelial tissues [2, 3]. Nectins form a complex network of trans-interactions at various specific intercellular junctions. The most distal Ig-domain (V-domain) contains a canonical binding site that defines ligand specificity [4]. Upon trans-interaction with ligands, nectins accumulate at cell contacts and recruit junctional proteins to organize junctions. In particular, nectin-1 (CD111) participates in the establishment of adherence junctions (AJ) of epithelial cells and synapses of neurons [1, 5].

Several nectins are used as entry receptors by different human and animal viruses. Nectin-1 is the most ubiquitous receptor for herpesviruses including herpes simplex viruses (HSV-1 and HSV-2), porcine pseudorabies virus (PRV), bovine herpes virus type 1 (BHV-1), and simian B virus[6–8]. Nectin-2 (CD112) is used by HSV-2, PVR and some laboratory strains of HSV-1 [9]. Necl-5 (CD155) is the original poliovirus receptor [10] and is also used by PVR and BHV-1[6]. Finally, nectin-4 is a receptor for several morbilliviruses, including measles virus in humans [11, 12]. Herpesviruses bind nectins via the envelope glycoprotein D (gD), present in most alpha-herpesviruses [13, 14]. Nectin binding triggers conformational changes in gD that activate the regulatory heterodimer gH/gL and the fusion protein gB to allow membrane fusion and delivery of the viral genome in the cytoplasm of the target cell [15–17]. The binding site for gD on nectin-1 largely overlaps the canonical adhesive site [18–20]. Thus, HSV gD directly competes with ligand binding and inhibits nectin-1 adhesion at cell contacts [21, 22].

A role for nectins in regulating innate immunity has been uncovered more recently [23–26]. Natural Killer (NK) cell activity is regulated by the integration of both activating and inhibitory signals acquired by receptors at the immunological synapse [27]. Several nectins act as ligands for NK cell receptors to promote attachment and regulate activation of NK cells [28–33]. Nectin-2 and CD155 bind and regulate the potent activating receptor CD226 (DNAM-1) [34]. Co-activation by CD226 may play an important role in NK cell-defense against cancer since nectin-2 and CD155 are often overexpressed on tumor cells [34, 35]. Interestingly, the inhibitory receptor TIGIT also binds CD155 and nectin-2 and counteracts the effects of CD226 [36]. Recently a novel receptor for nectin-2 (CD112) was identified on T cells and named CD112R (or PVRIG) [37]. CD112R specifically binds nectin-2 with high affinity and blockade of CD112R increased NK cell anti-tumor response [37–39]. The third and less-characterized receptor from this group, CD96 (TACTILE), binds CD155, but not nectin-2 [28]. CD96 was first identified as an antigen of activated T cells and is distantly related to nectins [40]. Its short cytoplasmic tail contains an immunoreceptor tyrosine-based inhibition motif (ITIM), as well as a Proline-rich sequence that may play a signaling role in activation [41]. Determining the role of CD96 in NK cell activation or inhibition has proven notoriously difficult [41]. Fuchs *et al* showed that cytotoxicity of human polyclonal NK cell lines was enhanced in the presence of anti-CD96 antibody [28]. This suggested that engagement of human CD96 favored NK cell activation rather than inhibition. In mice however, strong evidence indicate that mCD96 inhibits anti-tumor NK cell activity, mostly by limiting IFNγ production [41, 42]. Beside mCD155, mCD96 binds mNectin-1, albeit less efficiently [31]. However, the actual role of mNectin-1 in murine NK cell activation or inhibition has not been determined. Altogether CD96, CD226 and TIGIT form a balanced regulatory system that controls NK cell activation by interacting with CD155, nectin-2 and nectin-1 [25, 26, 41].

NK cells play major roles against tumors and infected cells. NK cells are critical in controlling infections by viruses, which escape CTL defenses by down regulating MHC-1 molecule, in particular herpesviruses [43]. Consequently a number of natural killer cell deficiencies (NKD) result in increased risk and severity of infection by herpesviruses [43, 44]. Furthermore, these viruses have evolved numerous strategies to escape NK cells, such as down-regulating ligands for activating receptors on NK cells, while expressing viral mimics of ligands for inhibitory receptors [45, 46]. Most, if not all herpesviruses target ligands of NKG2D, by preventing their expression at the cell surface [45]. Human cytomegalovirus (HCMV) proteins UL141 and US2 cooperate to downregulate nectin-2 and CD155 from the cell surface [47–49]. Neurotropic alpha-herpesviruses that use nectins as entry receptors can directly use the entry glycoprotein gD to down regulate these nectins from the surface of infected cells. For instance, PRV gD induces down-regulation of nectin-2, but not CD155, thereby reducing DNAM-1 binding and NK cell killing [50]. HSV-2 can use nectin-2 as a receptor [9] and HSV-2 gD expression also down-regulates nectin-2 to prevent DNAM-1 binding and NK cell killing [50]. Nectin-1 is rapidly downregulated from the surface of infected cells [51, 52]. Interestingly, cell surface expression of gD also induces down regulation of nectin-1 from the surface of adjacent cells [53]. Similar to nectin-1 natural ligands, HSV gD binds to the canonical adhesive site of nectin-1 [4, 18, 54], however the mechanism leading internalization rather than adhesion remains unclear [18, 53].

Finally, both nectin-1 and CD96 have been shown to play a role in human development [2, 55]. Nectin-1 deficiency is linked to craniofacial, skin and digits abnormalities in patients affected by cleft lip/palate ectodermal dysplasia type 1 (CLPED1) (MIM #225060) [56]. These symptoms are likely caused by a defect in cell-cell adhesion during development. In genetic knock-out mice, the lack of nectin-1 results in microphthalmia and dental abnormalities [57, 58]. Interestingly, mutations in human CD96 have been linked to a complex developmental defect named *C (Opitz trigonocephaly) Syndrome* [55]. This severe C syndrome (MIM #211750) comprises multiple craniofacial abnormalities, visceral, skin and limb defects, as well as psychomotor retardation. The effect of CD96 deficiency on the immune system of these patients was not investigated [55]. In contrast CD96-/- mice have increased inflammatory response and resistance to carcinogenesis, but no described developmental defects [42]. Human CLPED1 and C syndromes are complex but may in part result from inadequate cell adhesion caused by the lack of interaction between nectin-1 and CD96 during development.

In this study, we describe the binding of human nectin-1 to CD96. We found that human nectin-1 and CD96 interact with micromolar affinity and we located the binding site for hCD96 on the nectin-1 V-domain, which contains the canonical binding site used for nectin trans-interactions and HSV attachment. We also show that a functional GFP-tagged nectin-1, which was expressed at the surface of K562 cells, increased the susceptibility of these targets to killing by NK-92 cells.

## Material and methods

### Cells, proteins and virus

Human NK-92 cells [59] were maintained in Myelocult medium (STEMCELL technologies) supplemented with hIL-2 (100 U/ml) and penicillin/streptomycin. NK-sensitive human erythroleukemia K562 cells [60] were maintained in R10 medium (RPMI supplemented with 10% heat-inactivated fetal calf serum, 2 mM L-glutamine, 1 mM sodium pyruvate, 1X MEM non-essential amino acids (GIBCO), 0.01 M HEPES, and penicillin/streptomycin. Proteins such as nectin-1(143t), nectin-1(346t), gD(306t) were produced in Sf9 cells infected with recombinant baculovirus and purified as previously described [13, 61, 62]. The fusion protein nectin-1(143t)-MBP (Maltose-binding Protein) was produced in E. coli and purified as previously described [18]. The reporter virus HSV-1 KOStk12 was generously obtained from P.G. Spear [9]. Viruses were produced and titered on Vero cells, and purified as previously described [63].

### Cloning human CD96 from NK-92 cells

Total RNA from 10^7^ NK-92 cells was purified using an RNeasy kit (QIAgen) and complementary DNA was obtained by reverse transcription and polymerase chain reaction (PCR) using an Omniscript kit (QIAgen) according to the manufacturer’s instructions. Synthetic primers were designed from available sequences in the NCBI nucleotide database and were used for amplification of the full-length open reading frame (ORF) or the CD96 ectodomain for baculovirus expression. To amplify and clone the full-length ORF, the forward primer CD96FLFwd (GCCGGATCC**ATG**GAGAAAAAATGGAAATACTGTG) added a BamH1 restriction site directly upstream of the initial ATG and the reverse primer CD96FLRev (GCCT**CTA**GAGGGTCTCCATCTCATG) added an XbaI site overlapping the stop codon. To amplify, tag and clone the CD96 ectodomain, the forward primer CD96BacFwd (GCCGGATCCA**GTT**TGGGAAAAAACAGTCAACACAG) added a BamH1 site upstream of codon GTT encoding Val22 of the open reading frame, to remove the native signal peptide. The reverse primer CD96BacRev (GCCGAATTC**TTA***ATGATGATGATGATGATG*ATCTTTGGGCT TATTGACCAC) added an EcoR1 restriction site immediately downstream of a stop codon, which follows six histidine codons positioned after Lysine 516. The amplified product was gel purified using GeneClean (MP Biomedical) and inserted in the corresponding restriction sites of plasmid pVTBac [64] to generate plasmid pCM6.

### Expression of soluble CD96 ectodomain in recombinant baculovirus

In plasmid pCM6 the mellitin signal sequence provided by pVTBac replaced the CD96 signal peptide sequence to ensure efficient secretion of the CD96t ectodomain [64]. The generation of recombinant baculovirus has been published previously [65, 66]. Briefly, plasmid pCM6 was co-transfected with Baculogold DNA (Pharmingen) into Sf9 cells. Recombinant baculoviruses were purified through two rounds of plaque selection on Sf9 cell monolayers. Expression of CD96t was assessed by Western blotting using an anti-tetra-His tag antibody (QIAgen). The recombinant baculovirus was named Bac-hCD96(516t) and the recombinant protein was designated hCD96(516t), or hCD96t for short.

For protein production, Sf9 cells in suspension cultures were infected with bac-hCD96(516t) at a multiplicity of infection (MOI) of 4 plaque forming units (pfu) per cell. After 48 h cells were removed by centrifugation at 2000 x g, at 4°C for 30 min. The supernatant was filtered through a 0.22 μm membrane, and dialyzed against PBS using a 10 kDa cut-off membrane (SpectraPor). About two ml of Ni-NTA resin (QIAgen) pre-equilibrated with PBS were added per 1-liter culture and incubated O/N at 4°C on a rotary shaker. The resin was pelleted at 500 rpm, for 10 min at 4°C, transferred to a column and washed with PBS. The bound protein was eluted with increasing concentrations of imidazole (10 mM, 25 mM, 50 mM, 250 mM and 500 mM) in PBS. Based on purity, 100 or 250 mM imidazole fraction were dialyzed against PBS and concentrated through a 10 kDa molecular weight cut-off centrifugation membrane (Millipore). Protein analysis was performed by SDS-PAGE followed by silver staining or western blotting using an anti-tetra-His antibody (QIAgen) as described previously [13, 66].

### Enzyme-linked immunosorbent assay (ELISA)

Nectin-1(143t)-MPB was purified from bacterial extract as described previously [18]. Purified Nectin-1(143t)-MPB was diluted to 10 μg/ml in PBS and used to coat 96-well ELISA plates overnight at 4°C. Control wells included only milk proteins. Plates were washed with 0.1% Tween 20 in PBS (PBS-Tween) and incubated in blocking solution (PBS-Tween with 5% milk) for 1 h at room temperature (RT). Plates were washed with PBS-Tween and incubated with various concentrations of hCD96(516t) in blocking solution for 3 h at RT. Plates were washed with PBS-Tween and incubated in blocking solution containing anti-tetra-His antibody (QIAgen) at 0.2 μg/ml for 1 h at RT. After being washed with PBS-Tween, the plates were incubated with horseradish peroxidase-conjugated secondary anti-mouse Ig antibody at 0.2 μg/ml in blocking solution for 30 min at RT. Plates were then washed with PBS-Tween and with 20 mM citrate buffer (pH 4.5). The horseradish peroxidase substrate [2,2′-azinobis(3-ethylbenzthiazolinesulfonic acid); Moss, Inc.] in citrate buffer (pH 4.5) was added, and the absorbance at 405 nm was read with a microtiter plate reader (Bio-Tek).

### Surface plasmon resonance (SPR)

SPR experiments were performed on a BIAcore 3000 optical biosensor, at 25°C following the protocol previously described [61, 67]. The running buffer was HBS-EP (10 mM HEPES, 150 mM NaCl, 3 mM EDTA, 0.005% polysorbate 20), pH 7.4. Approximately 2000 RU of purified nectin-1(346t) or nectin-1(143t) were coupled to the surface of flow cell 2 (Fc2) of a CM5 chip (BIAcore) via primary amines using a BIAcore X optical biosensor. Fc1 was mock treated without the addition of protein to serve as a control surface for non-specific interactions. Soluble hCD96t was serially diluted in HBS-EP and each sample was injected for 2 min to monitor association. Then the sample was replaced by HBS-EP flow, and the dissociation was monitored for 2 min. The flow path was set to include both Fc1 and Fc2, the flow rate was 50 μl/min and the data collection rate was set to high (5 measurements /min). When necessary, regeneration of the nectin-1 surface was achieved by injecting brief pulses of 0.2 M Na_2_CO_3_, pH 11.5 until the response signal returned to baseline. Sensorgrams were corrected for non-specific binding and refractive index changes by subtracting the control sensorgram (Fc1) from the nectin-1 surface sensorgram (Fc2). Data were analyzed with BIAevaluation software, version 3.0 (5). Model curve fitting was done by using a 1:1 Langmuir interaction with drifting baseline, unless specified otherwise.

### Expression of GFP-nectin-1 in K562 cells

Human erythroleukemia K562 cells were electroporated with plasmid pCK495 to express GFP-nectin-1 [53] using an AMAXA Cellfector. One million cells were resuspended with 3 μg plasmid in Amaxa solution V and electroporated according to the manufacturer’s instructions. To establish stable transfectants, cells were maintained in culture medium in the presence of 0.5 mg/ml G418. Single cell limiting dilution was performed to obtain clonal populations. Since GFP-nectin-1 remained heterogeneous in these populations, enrichment of positive cells was achieved by fluorescence activated cell sorting (FACS) based on GFP expression (UPENN Flow Cytometry and Cell Sorting Laboratory). Sorted clone #11 was used, unless mentioned otherwise, and referred to as K562-N1G cells. Positive cells were maintained in growth medium in the presence of 0.5 mg/ml G418 and monitored regularly for GFP fluorescence.

### Immunofluorescence assay

K562 cells were fixed with paraformaldehyde at a final concentration of 3% about 48 h post electroporation with plasmid pCK495 [53]. Expression and localization of GFP-nectin-1 was assessed by observing GFP fluorescence using a Nikon Eclipse E600 equipped with a Hamamatsu C4742-95 camera.

### Flow cytometry

Immunodetection of nectin-1-GFP on transfected K562 cells was performed using anti-nectin-1 monoclonal antibody CK41 [68], which was directly coupled to phycoerythrin (PE). Typically, 0.5 x10^6^ cells were stained in 50 μl FACS buffer (PBS, 3% FCS, 0.01% sodium azide) containing 5 μg/ml CK41-PE for 30 min at RT. Cells were washed by the addition of 1 ml FACS buffer, centrifuged at 750 x g for 5 min and resuspended in FACS buffer containing 3% paraformaldehyde. Cells were analyzed on a Becton Dickinson LSR II flow cytometer. For GFP expression, cells were simply washed once in FACS buffer and fixed as above. Immunodetection of nectin-3 and nectin-1 on K562 and NK-92 cells was performed using monoclonal antibodies (Mabs) N3.14.2 (Millipore) and CK41 [68] respectively. Typically, 0.5 x10^6^ cells were stained in 100 μl FACS buffer (PBS, 3% FCS, 0.01% sodium azide) containing 10 μg/ml anti-nectin Mab for 1 h on ice. As an isotype control, a mouse anti-FLAG Mab (Sigma Aldrich) was used in parallel. Cells were washed by the addition of 1 ml FACS buffer, centrifuged at 750 x g for 5 min, resuspended in 100 μl FACS buffer containing 4 μg/ml CF^TM^488A-conjugated anti-mouse antibody (Sigma Aldrich) and incubated on ice for 30 min. Cells were washed by adding 1 ml FACS buffer, centrifuged at 750 x g for 5 min and resuspended in FACS buffer with 3% paraformaldehyde. Cells were analyzed on a Becton-Dickinson FACSCelesta flow cytometer.

### HSV entry assay

Cells were seeded in a 96 well culture plate at 5x10^4^ cells/well in 50 μl growth medium. The reporter virus HSV-1 KOS tk12, carrying the *lacZ* reporter gene, was added at the indicated multiplicity of infection (MOI) to a final volume of 100 μl/well. Infection proceeded at 37°C for 12 h, before cells were lysed by the addition of NP40 to a final concentration of 0.5%. A 50-μl volume of cell lysate was mixed with an equal volume of β-galactosidase substrate (chlorophenol red-β-d-galactopyranoside; Roche). The level of entry was monitored by reading absorbance at 570 nm for 50 min to record enzymatic activity, which is expressed as the change in absorbance per hour [69].

### Cytotoxicity assay

NK cell cytotoxicity was assessed using a standard chromium (^51^Cr) release assay [70, 71]. NK-92 cells were used as effectors. K562 and K562-N1G cells were loaded with ^51^Cr for 1h at 37°C, washed with medium and resuspended in R10 medium. Assays were performed in triplicates in 96-well round-bottom plates with 1x10^4^ target cells mixed with NK-92 effector cells at the indicated ratio in a total volume of 200 μl. After 4h incubation at 37C, cells were centrifuged at 300g and free ^51^Cr was in quantified in 100 μl co-culture supernatant using a TopCount XL counter (PerkinElmer). Percent lysis was determined as (average experimental release–average spontaneous release) x 100 / (average total counts–average spontaneous release). Spontaneous release values are obtained from culture of target cells in the absence of effectors. Total counts are determined after detergent lysis of target cells cultured under experimental conditions in the absence of effectors [71].

### Statistical analysis

Statistical analysis was performed based on the mean and the SEM of at least three independent replicates, using a one-way ANOVA test.

## Results

### Cloning, sequence and production of human CD96 protein

Because mouse nectin-1 is known to interact with mouse CD96 [31], we tested whether nectin-1 could interact with human CD96. In order to produce recombinant human CD96 protein in the baculovirus expression system, we first isolated a human CD96 cDNA from NK-92 cells. Expression of CD96 by NK-92 cells has been previously characterized in detail [28]. DNA sequencing identified the CD96 cDNA isolated from NK-92 cells as corresponding to the variant 2 isoform generated by alternative splicing. The NK-92 hCD96 sequence was identical to the Genbank sequence NM_005816.4 with a single nucleotide substitution A170G, leading to a Q57R change in the protein and a three nucleotide deletion (CAG 1322–1324) replacing Ser/Val (441/442) with—/Phe. The numbering of residues in this article will follow the Genbank sequence NM_005816.4. In all previously tested human tissues, variant 2 mRNA expression is predominant as compared to variant 1 [72]. The expression of the longer variant 1 in NK-92 cells was not investigated in this study.

**Fig 1:**
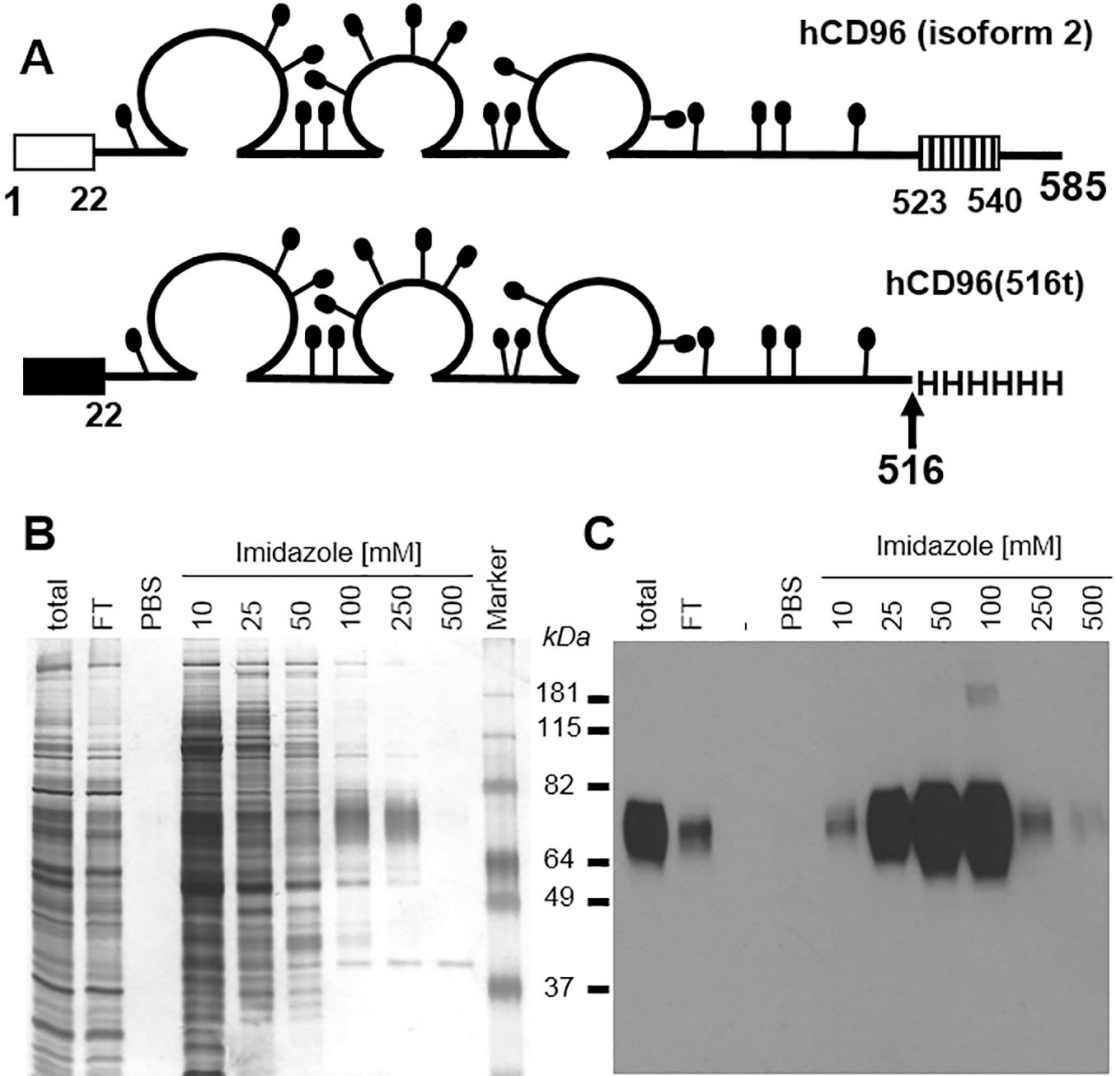
Production of recombinant human CD96. (A) Schematic representation of human CD96 protein, the 585 amino acid long isoform 2 is represented with residues numbered from methionine 1. The open box represents the signal peptide and the hatched box represents the transmembrane domain. The putative N-linked carbohydrates are shown as black lollipops. In the baculovirus construct, the mellitin signal peptide (black box) replaced the natural signal peptide (amino acids 1 to 21) from CD96. Human CD96 was truncated after lysine 516, and six histidine residues were added at the C terminus of hCD96(516t). (B) Silver stain and (C) western blot analysis of baculovirus-produced hCD96t after nickel chromatography purification and SDS-PAGE under denaturing and reducing conditions. Concentrations of imidazole used to elute proteins from the Ni-NTA-agarose column are indicated. FT = flow through. PBS indicates extensive PBS wash of the column before elution. Sizes of the molecular weight marker are indicated in kilodaltons. Anti-tetraHis Mab (QIAgen) was used to detect tagged hCD96t by western blot (C).

To obtain large amounts of pure hCD96 ectodomain, a recombinant baculovirus was engineered to express hCD96 truncated at amino acid 516, before the transmembrane domain (Fig 1A). The recombinant protein was tagged at the C-terminus with 6-histidines and purified from the insect cell culture medium using a single-step nickel affinity column. When recombinant hCD96t was eluted using increasing concentrations of imidazole, the purest protein was found in the 100 and 250 mM imidazole fractions, as determined by silver stain of total proteins and CD96 western blotting (Fig 1B and 1C). The apparent 78 kDa size of hCD96t by SDS PAGE, corresponds to the calculated molecular weight of the polypeptide (58.5 kDa) with an additional 15.5 kDa attributable to carbohydrates at the potential 17 N-glycosylation consensus sites. The thickness of the band on the gel is typical of glycosylated proteins [13]. The purified hCD96t was also detected by ELISA using a specific anti-CD96 monoclonal antibody (Clone 3H*, Abnova) (data not shown). Thus, the ectodomain from isoform 2 of hCD96 can be cloned from NK-92 cells and purified for biochemical assays.

### Interaction between hCD96t and nectin-1 *in vitro*

**Fig 2:**
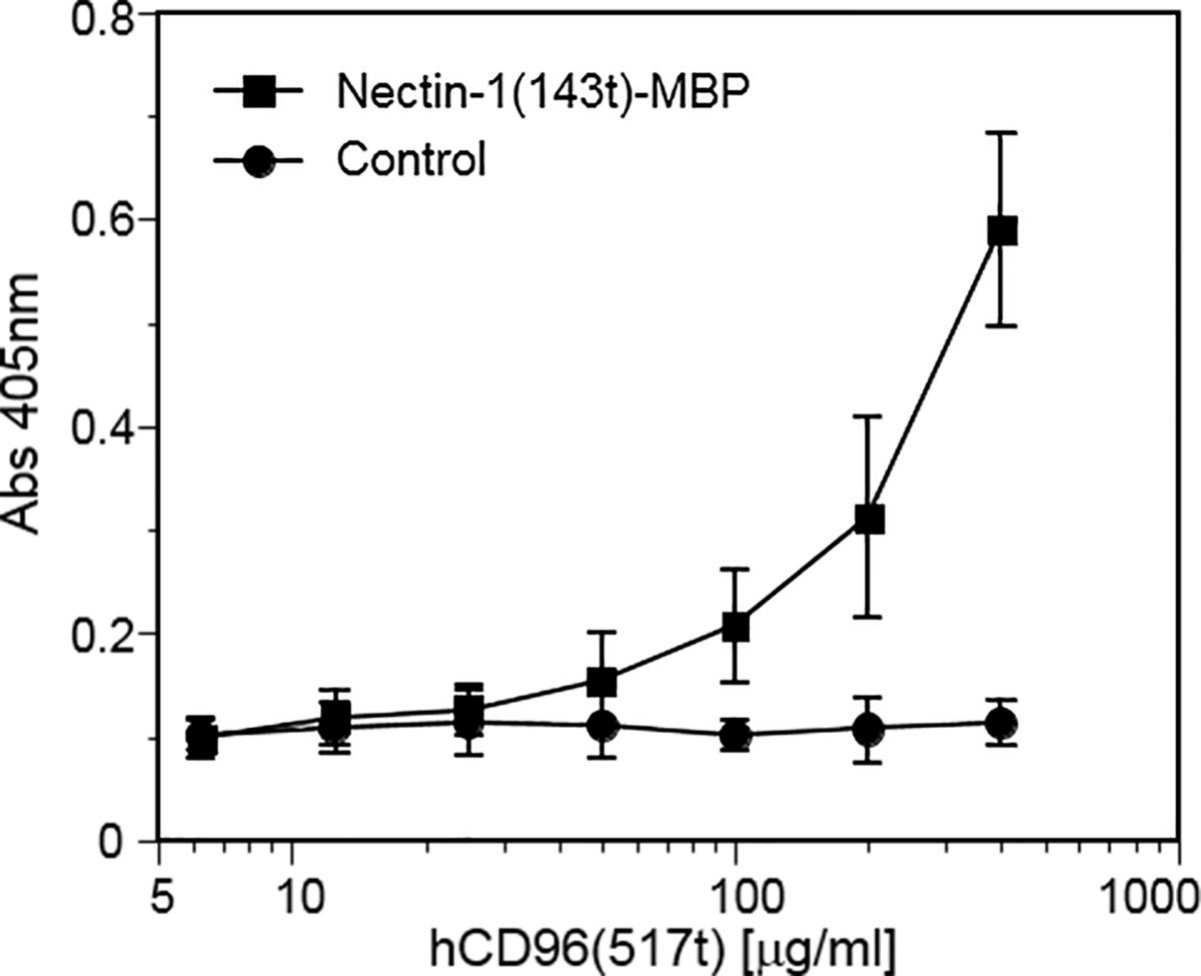
Interaction between human CD96 and nectin-1 V-domain by ELISA. Plates were coated with Nectin-1(143t)-MBP at 10 μg/ml, or mock treated (milk protein control). Increasing concentrations of hCD96t were added and bound hCD96t was detected using an anti-tetraHis tag Mab (Qiagen), followed by secondary antibody and substrate. Absorbance was read at 405 nm. Average values ± one standard deviation of four independent experiments are shown.

**Fig 3:**
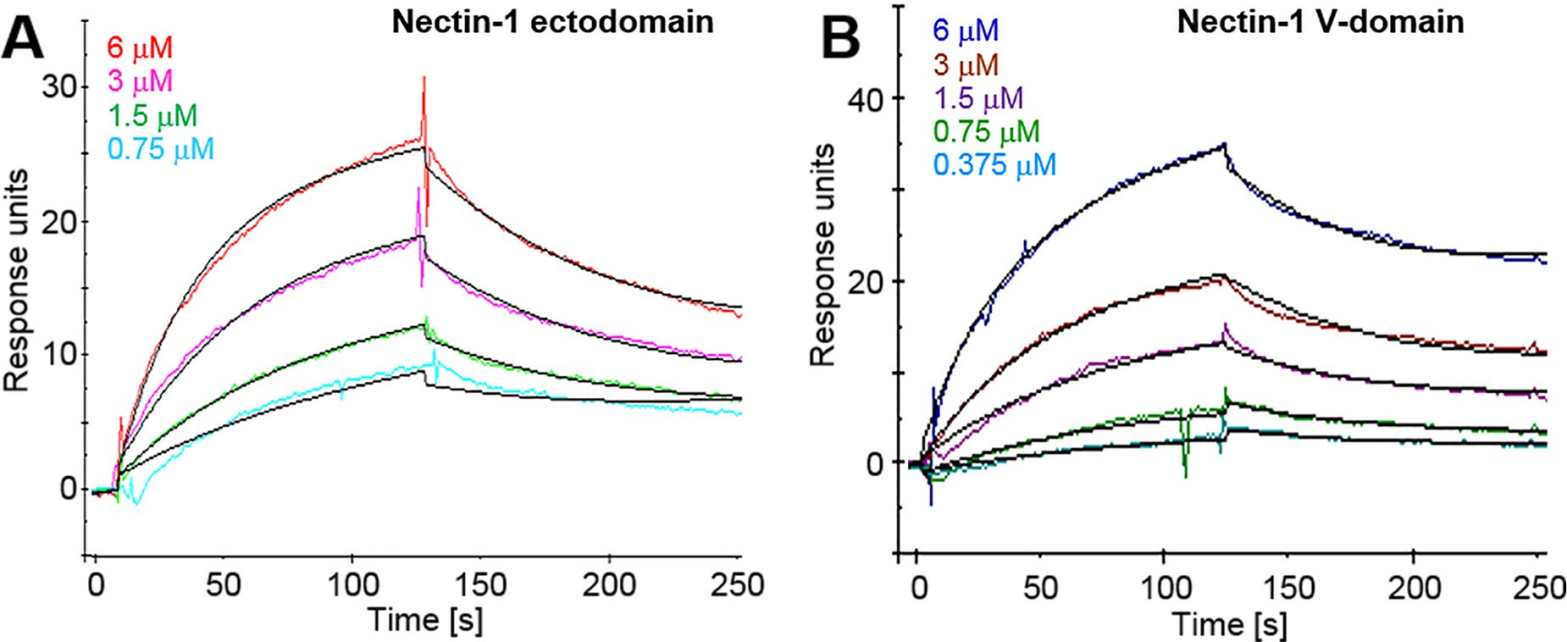
Surface Plasmon Resonance analysis of the interaction between human CD96 and nectin-1 in real time. Nectin-1(346t) (A) or nectin-1(143t) (B) were immobilized on a CM5 biosensor chip. Various concentrations of hCD96t were flowed over the chip for 2 min (association) and then replaced by buffer for another 2 min (dissociation). Sensorgrams of corrected data are represented after the subtraction of signal from the control flow cell without immobilized nectin-1. Data points were collected at 5 Hz and are represented in color for each of the indicated concentrations of hCD96t. The black lines show the best fit obtained after global fitting with the BIAevaluation 3.0 software using a Langmuir 1:1 model with drifting baseline.

Previous studies show that hCD96 binds CD155 but no other known nectins, while mCD96 has been shown to also interact with mNectin-1 [28, 31]. The high degree of genetic conservation of nectin-1 (>96% amino acid identity between human and mouse) suggests conservation of functions. Therefore we assessed the direct binding of hCD96t to nectin-1. The consensus binding site of nectins is located in the distal V-domain, thus we used a recombinant nectin-1 V-domain produced in E. coli [18] as a ligand in ELISA (Fig 2). A dose-dependent specific binding of hCD96t to purified hNectin-1(143t)-MBP was observed. Since bacterially produced proteins were not glycosylated, N-glycosylation of nectin-1(143t)-MBP was not essential for binding to CD96. To confirm the ligand specificity and obtain quantitative data on the binding kinetics, we used a surface plasmon resonance (SPR) approach to analyze binding in real-time. Since immunodetection is dispensable in SPR assays, we could use histidine-tagged forms of nectin-1 produced in insect cells as ligands. Binding to the nectin-1 full ectodomain (Fig 3A) and V-domain (Fig 3B) was detected at various concentrations of hCD96t. Affinity and kinetics constants were obtained after fitting data to a 1:1 binding model (Fig 3 and Table 1). The affinity constant K_D_ is similar for both forms of nectin-1 suggesting that the binding site for hCD96t is located on the V-domain. The rates of complex formation (k_on_) and dissociation (k_off_) were also comparable between the nectin-1 ectodomain and V-domain. These SPR data suggested that the binding site for CD96 was entirely located on the V-domain of nectin-1, similar to the canonical site used for homophilic trans-interaction and for binding of HSV gD [18]. The affinity of hCD96t for nectin-1 (ectodomain and V-domain) is in the micromolar range (2.34 and 3.22 μM respectively). Interestingly, the affinity of HSV gD for nectin-1 was in the same range, when determined under similar experimental conditions (Table 1) [61]. However, the affinity for of nectin-1 for itself (trans-dimerization) is higher (17.5 μM) [4]. In this comprehensive study by Harrison et al, proteins produced in mammalian cells were used to measure affinities between nectins [4]. Here, purified proteins were produced in insect cells that only allow partial glycosylation. One cannot exclude that glycosylation may influence the affinity of nectin complexes following homophilic and heterophilic trans-interactions. However, glycosylation is not required for high affinity binding since structural determination of nectin-ligand complexes could be determined with bacterially produced recombinant proteins [73–75]. Overall, our data are consistent with hCD96 being a ligand for nectin-1 with properties similar to other cellular and viral ligands.

**Table 1:** Affinity and kinetics constants of CD96(517t) ectodomain to nectin-1(346t) ectodomain and nectin-1(143t) V-domain determined by SPR.

	Nectin1(346t)		Nectin-1(143t)	
	CD96(517t)^a^	gD(306t)^b^	CD96(517t)^a^	gD(306t)^b^
K_D_ [10^−6^ M]	2.43 ± 0.24	3.2	3.22 ± 0.59	1.2
k_on_ [10^3^ s^-1^M^-1^]	3.9 ± 1.48	2.2	3.2 ± 1.26	9
k_off_ [10^−2^ s^-1^]	0.94 ± 0.3	0.7	1.2 ± 0.14	1.1

^a^ means ± one standard deviation of values from 3 independent experiments.

^b^ values reported by Krummenacher et al, 1999.

### Expression of nectin-1 in K562 cells

**Fig 4:**
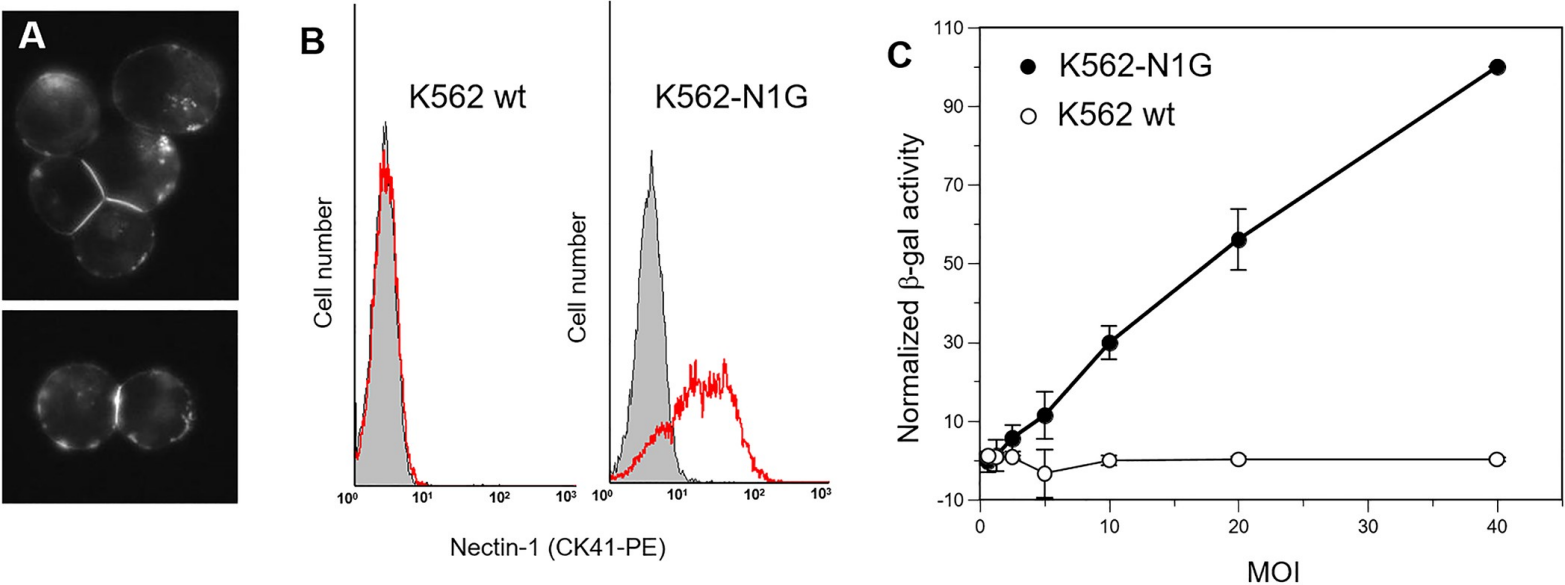
Expression of GFP-nectin-1 in human K562 cells. (A) K562 cells transiently transfected with plasmid pCK495 express nectin-1 with GFP tagged at the N-terminus. GFP-nectin-1 is expressed at the cell surface and accumulates at cell-cell contacts. Images of GFP fluorescence, magnification 40x. (B) Flow cytometry analysis of K562 cells stably transfected to express GFP-nectin-1. Cells were stained with PE-tagged anti-nectin-1 antibody CK41. Left histogram: K562 cells stained with CK41-PE (red line) are compared to control (gray shade). Right panel: cells from clone K562-N1G #11 expressing GFP-nectin-1 were stained with CK41-PE (red lines vs control shaded in gray). (C) Wild type K562 cells and K562-N1G cells were exposed to *lacZ* reporter virus HSV-1 KOStk12 at the indicated MOI. Activity of the virus-encoded beta-galactosidase was recorded as the change of OD_570nm_ over time to reflect virus entry. Values from at least two independent experiments were normalized to the highest value (K562-N1G cells at MOI = 40) set at 100% and averaged. Error bars indicate standard deviations across independent experiments.

To determine the functional contribution of nectin-1 to cytotoxic killing by human NK cells, we first engineered human erythroleukemia K562 cells to express human nectin-1. K562 cells are well-characterized targets in human NK cell cytotoxicity assays and do not express detectable levels of nectin-1 by FACS (Fig 4B). We have shown previously that human nectin-1 fused with GFP at the N-terminus is functional for cell adhesion and HSV infection in mouse melanoma B78H1 cells [53]. When expressed in K562 cells, GFP-nectin-1 readily accumulated at cell contacts (Fig 4A). This accumulation confirms the correct processing of GFP-nectin-1 in K562 cells and its ability to trans-interact with a ligand at cell contacts. A similar accumulation of GFP-nectin-1 at cell contacts was observed in a functional adhesion assay using B78H1 cells [52]. We then used limited dilutions of stably transfected K562 cells expressing GFP nectin-1 (K562-N1G) to obtain clonal lines. Positive cells were then sorted by flow cytometry based on GFP fluorescence to select for high GFP-nectin-1-levels and limit heterogeneity of expression in the transfected cell line. K562-N1G clone #11 exhibited stable expression of GFP-nectin-1 and was selected for functional assays. These K562-N1G cells expressed high levels of surface nectin-1, as tested by immunodetection with anti-nectin-1 Mab CK41 (Fig 4B) [68]. Nectin-1 levels at the surface of K562-N1G cells appear to be within physiological range. Higher expression was reported in keratinocytes (HaCat) and neuroblastoma cells (SY5Y) [68, 76]. Many cell lines surveyed with Mab CK41 under similar conditions showed detectable, but lower levels of nectin-1 than K562-N1G cells [69]. To confirm that GFP-nectin-1 was functional in K562-N1G cells, we used an HSV infection assay [69]. Using different cell lines, we and others have shown that the amount of available nectin-1 correlated with susceptibility to infection [69, 77]. In the absence of nectin-1, wild type K562 cells were resistant to infection while K562-N1G cells became infected (Fig 4C). Overall, these data demonstrated that GFP-nectin-1 was functional as an adhesion molecule and as a viral receptor at the surface of K562-N1G cells.

### Effect of nectin-1 on the susceptibility to NK cell cytotoxicity

**Fig 5:**
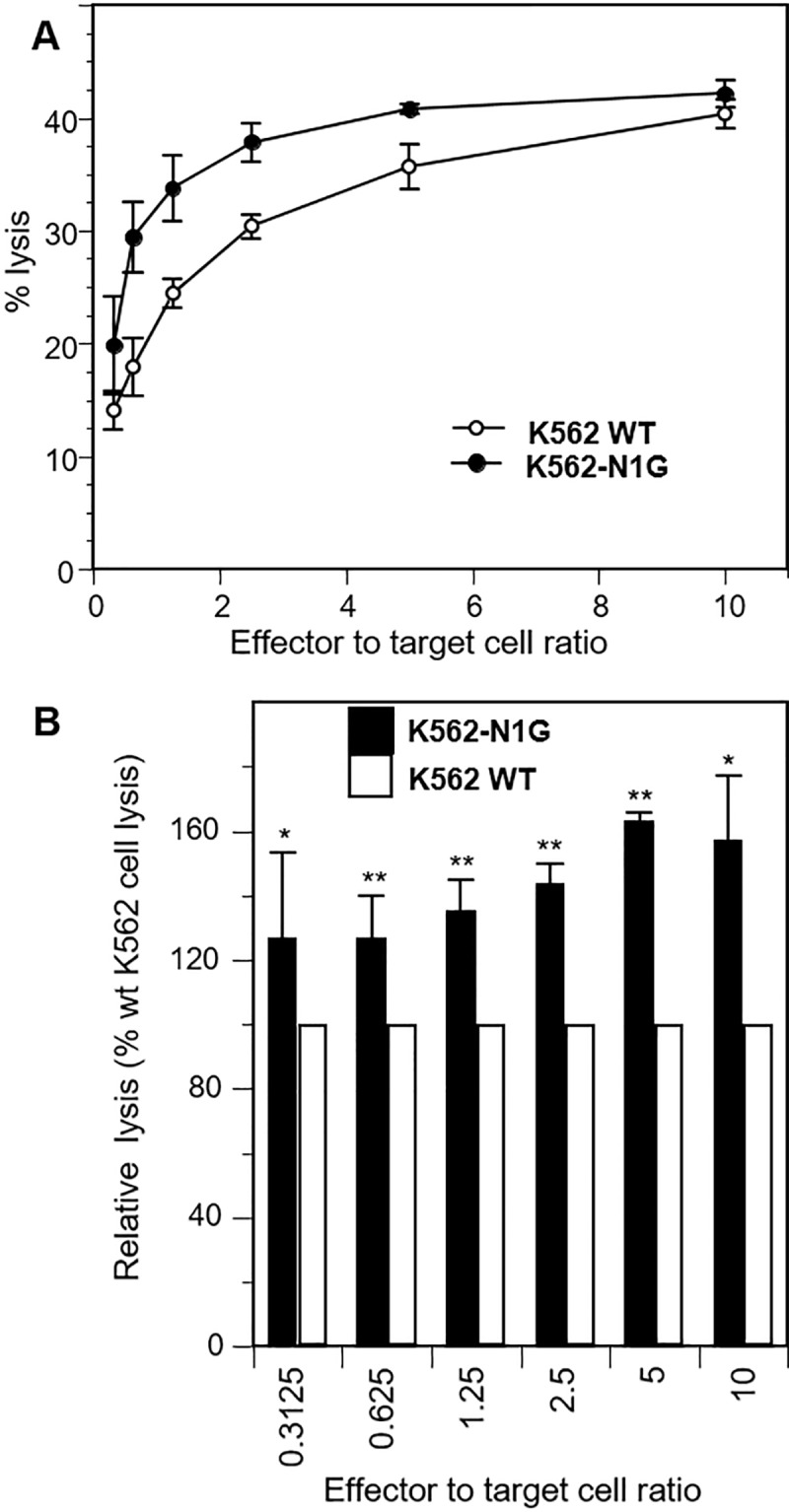
Effect on nectin-1 on susceptibility to NK cell cytotoxicity. Wild type K562 cells or K562-N1G cells, which expresses GFP-nectin-1 were labeled with ^51^Cr and mixed with effector NK-92 cells at the indicated ratio and incubated for 4 h. Release of ^51^Cr is measured and % specific release is compared to K562 and K562-N1G in the absence of NK cells (0% lysis) or lysed with detergent (100% lysis). (A) Data from a representative experiment shows average of triplicate measurements ± one standard deviation. (B) Combined data from four independent experiments show the relative killing of K562-NG1 compared to wild type K562 (normalized to 100%) at different effector to target ratios. Averages values ± one standard deviation are shown. Significance of differences between cell types at each effector to target ratio was determined by one way ANOVA (**P<0.01; *P<0.05).

To determine the role of nectin-1 in the susceptibility to NK cell cytotoxicity, naïve or stably transfected K562 cells expressing GFP-nectin-1 (K562-N1G cells) were used as targets for NK-92 cells. NK-92 cells were initially derived from the peripheral blood of a patient with non-Hodgkin’s lymphoma and have strong cytotoxicity against K562 and Daudi cells [59]. They are negative for CD4 and CD8 but have been shown to express CD96 [28, 59]. A standard 4 hour chromium-release assay [70, 71] was performed using effector to target ratios from 10 to 0.31 (Fig 5). As expected, mock-transfected wild type K562 cells were efficiently killed by NK-92 cells. Importantly, K562-N1G cells which express human nectin-1 on their surface were more susceptible to NK-92 cell cytotoxicity. The increase in susceptibility of K562-N1G cells to NK cell cytotoxic killing was most prominent at lower effector to target ratios between 0.62 and 2.5. Although it remained significant, this increase was less noticeable at higher effector to target ratios between 5 or 10 (Fig 5A). Overall these data indicate that human nectin-1 can play a positive role in increasing NK cell cytotoxicity when expressed on potential target cells.

The increased cytotoxicity of NK-92 cells could be caused in part by increased attachment to target K562 cells that express nectin-1. Adhesion of targets to NK cells could be enhanced by the interaction of nectin-1 with high affinity ligands such as nectin-3, or nectin-1 [4]. We used flow cytometry to measure expression of these nectins on target K562 and effector NK-92 cells using anti-nectin-1 (CK41) and anti-nectin-3 (N3.12.4) monoclonal antibodies (Mabs) (S1 Fig). For K562 cells, staining for nectin-1 and nectin-3 was only slightly above the isotype control (anti-FLAG Mab). These very low levels of expression indicated that nectin-1 and nectin-3 may play a limited role in recognition of wild type K562 cells by NK-92 cells. In contrast, NK-92 cells expressed high amounts of nectin-3 but no detectable amounts of nectin-1. Since nectin-3 has a high affinity for nectin-1 [4], it is possible that NK-92 cells attach more efficiently to target K562 cells that over express nectin-1.

## Discussion

The role of nectins and nectin-like molecules as key regulators of NK cell functions is now well established [23–26]. However, their effect on NK cells is complex as the same ligand (e.g. CD155, nectin-2) can both activate and inhibit NK cells though competing interaction with paired receptors CD226 and TIGIT [78]. The role of CD96 itself is less well defined and appears ambivalent as it has been shown to be an activator of human NK cells but an inhibitor of murine NK cells [24]. In both species CD96 interacts with CD155 with high affinity but only the mouse CD96 was previously shown to interact with nectin-1 [31]. Nectins are highly conserved adhesion molecules between species [2]. Even though nectin-like molecules and NK cell Ig-receptors are less conserved, the structural interface between all these molecules is remarkably maintained [4, 36, 79]. The canonical binding site for trans-interaction is located on the same side of the distal V-domain of nectins. By comparing the affinity of the hCD96 ectodomain to the nectin-1 ectodomain and V-domain (Table 1), we showed that the nectin-1 V-domain is sufficient for hCD96 binding with full affinity. Human CD155 binding to hCD96 is also mediated through the CD155 V-domain [28]. These observations are consistent with the use of the canonical nectin interface for binding hCD96. Reciprocally, the mCD96 V-domain is necessary for ligand binding [72]. However, structural differences in other domains affect CD96 binding. For instance, an 18 amino acid insertion in the second Ig-like domain of hCD96 isoform 1, which is already expressed at a 10-fold lower level than isoform 2 in all tissues tested, reduces binding to CD155 [72]. Since the hCD96 isoform 2 that we used here has a relatively low affinity for nectin-1, we did not test the rarer isoform 1, which presumably would bind with an even lower affinity. The interaction between human nectin-1 and hCD96 *in vitro* has a relatively low affinity compared to other interactions between nectins and NK cell Ig receptors (summarized in [1, 78]). The 2.4 μM affinity constant (K_D_) measured here is larger than the K_D_ of many nectin homophilic trans-interactions [1], suggesting that, on its own, CD96 may not interact with nectin-1 molecules already engaged in trans-interactions at cell junctions. The affinity of CD96 for nectin-1 is also lower than the affinity of CD96 for CD155 (37.6 nM) [80]. This suggests that CD155 may efficiently outcompete nectin-1 for CD96 binding. Indeed, K562 cells express high levels of CD155 [29, 81–84], which could potentially monopolize CD96 and limit the activity of nectin-1 in our cytotoxicity assay. Nectin-1 is not known to interact with CD155 [4] and we think it is unlikely that the presence of nectin-1 would directly affect expression of CD155 in transfected cells. However, the levels of CD155 in K562-N1G cells has not been measured. Overall, the affinity between various nectins and cognate NK cell receptors likely contributes to the regulation of NK cell activity, especially in a complex system where activating and inhibitory receptors share ligands on target cells [26, 78].

What are the implications of nectin-1 recognition on human NK cell functions? Here, we show that nectin-1 overexpression leads to increased susceptibility of K562 cells to NK-92 cell cytotoxicity. This is the first observation of the involvement of nectin-1 in NK cell activity. Nectin-1 is rather ubiquitously expressed in human tissues. As a cell adhesion molecules, it is mostly engaged in cell junctions and naturally inaccessible to NK cells. Only in pathological situations where junctions are disrupted would nectin-1 be exposed in sufficient quantities to be functionally recognized. For instance, intercellular junctions are frequently perturbed in metastatic tumors. In breast cancer metastatic tumors have increased expression of nectin-1 and nectin-2 [85]. In pancreatic tumors, overexpression of nectin-1 correlates with poor prognosis of the disease [86]. In such pathological situations, nectin-1 could be a target of choice for NK cells aiming at eliminating cancer cells. Whether the level of expression of nectin-1 in naturally occurring tumor cells affects their susceptibility to human NK cells remains to be determined. This is certainly the case in the model K562 cells where nectin-1 expression correlates with increased susceptibility to NK-92 cytotoxicity (Fig 4). However, the mode of action of nectin-1 is not elucidated. Two possible mechanisms could be envisaged. The first possibility is that nectin-1 might favor attachment of NK cells to their targets. The detection of nectin-3, a high affinity ligand for nectin-1, on NK-92 cells is consistent with a role in attachment. The second possibility is that nectin-1 might directly trigger an activator of NK cells. The ability of human nectin-1 to interact with CD96 opens the possibility that nectin-1 may have a more direct role in NK cell activation. However, the functional role of the nectin-1-CD96 interaction is not yet established. To understand how nectin-1 affects susceptibility of cells to NK cytotoxicity, it will be important to determine the contribution of a) nectin-3-mediated adhesion and b) CD96-mediated activation of NK cells.

What are the implications of nectin-1-mediated activation of NK cells for the innate immune defense against HSV? HSV infection affects the cell surface in many ways that can alter cell-cell recognition, which include modifying phospholipid composition [87], increasing expression of syndecans [88], decreasing expression of entry receptors [51, 89] and inducing membrane protrusions [90]. Since expression of nectin-1 increases cell susceptibility to NK cell cytotoxicity (Fig 4), the ability of HSV to interfere with nectin-1 function may reflect a novel immune evasion mechanism for this virus. HSV glycoprotein D (gD) is the viral ligand for nectin-1 and its binding allows virus entry into target cells [14, 16]. Interestingly, gD causes down-regulation of nectin-1 from the surface of infected cells [52] and therefore could potentially diminish recognition by NK cells. This viral behavior would be in line with that of PRV which uses its gD to down-regulate nectin-2 and protect infected cells from NK cell attacks [50]. Furthermore, HSV induces down-regulation of nectin-1 from the surface of adjacent cells [51, 53]. This effect of gD *in trans* prevents nectin-1 proteins to become exposed on these bystander cells. This could presumably further prevent stimulation of the NK cell response at the site of infection. Moreover, HSV gD binds to the canonical adhesive site on the V-domain of nectin-1 [18]. We have shown that gD binding prevents trans-interaction of nectin-1 homodimers and structural models suggest that it can sterically hinder interaction with nectin-3 at the canonical interface [21, 22]. hCD96 also interacts with the V-domain of nectin-1. Although a more detailed study is needed to determine if the nectin canonical adhesive site is used by hCD96, other nectins interact with Ig-like NK cell receptors through the same interface. For instance, the NK cell receptor TIGIT binds to the canonical site of its ligands nectin-2 and CD155 [73, 79]. This remarkable conservation of nectin interfaces suggests that nectin-1 binding to CD96 could be blocked by HSV gD. Moreover, since gD and CD96 have similar affinity for nectin-1 (Table 1) a competition between the two ligands may occur during infection. We previously showed that HSV gD could prevent nectin-1 adhesive function through down-regulation and ligand competition [22, 51, 89]. It can be envisaged that the same mechanisms will reduce the ability of nectin-1 to induce NK cell cytotoxicity during HSV infection. Clearly, further studies of human NK cells, *ex vivo* and in the context of infection, are needed to define the role of nectin-1, and CD96, in NK cell defense against HSV.

## Supporting information

S1 Fig Detection of nectin-1 and nectin-3 on K562 and NK-92 cells. Wild type K562 cells (top) and NK-92 cells (bottom) were analyzed for expression of nectin-1 (left) and nectin-3 (right) by flow cytometry. Staining with anti-nectin-1 CK41 Mab and anti-nectin-3 N3.12.4 Mab were compared to isotype control staining with anti-FLAG M2 Mab. The control histograms are colored gray, the nectin-1 histograms are colored blue and the nectin-3 histograms are colored red. The anti-nectin-1 CK41, which yielded no staining of K562 and NK-92 cells was active and able to stain control C10 cells in this experiment (not shown).
(TIF)
