# Supplementary figures and images for: Interaction between nectin-1 and the human natural killer cell receptor CD96
Source: PLoS One. 2019 Feb 13;14(2):e0212443. doi: 10.1371/journal.pone.0212443 (PMC6373967; doi:10.1371/journal.pone.0212443)

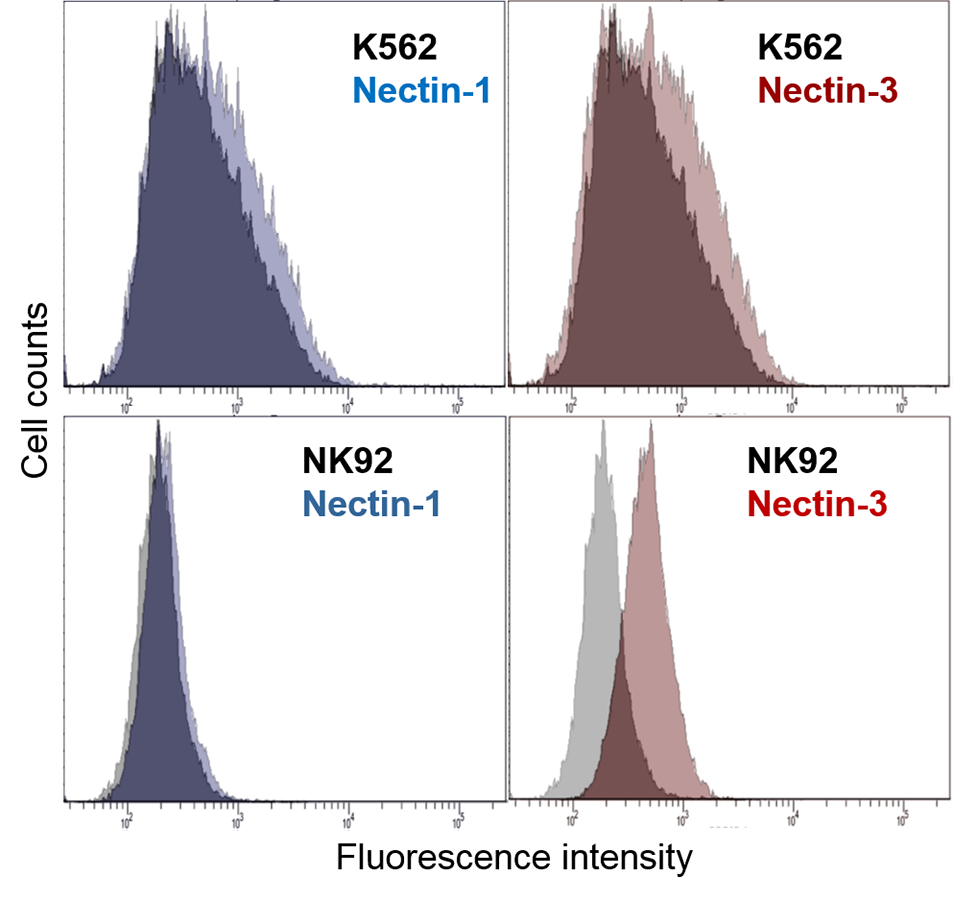

Supplement: S1 Fig — Wild type K562 cells (top) and NK-92 cells (bottom) were analyzed for expression of nectin-1 (left) and nectin-3 (right) by flow cytometry. Staining with anti-nectin-1 CK41 Mab and anti-nectin-3 N3.12.4 Mab were compared to isotype control staining with anti-FLAG M2 Mab. The control histograms are colored gray, the nectin-1 histograms are colored blue and the nectin-3 histograms are colored red. The anti-nectin-1 CK41, which yielded no staining of K562 and NK-92 cells was active and able to stain control C10 cells in this experiment (not shown). (TIF) [file pone.0212443.s001.tif]
